# Antagonistic pleiotropy in mice carrying a CAG repeat expansion in the range causing Huntington’s disease

**DOI:** 10.1038/s41598-018-37102-8

**Published:** 2019-01-10

**Authors:** A. J. Morton, E. A. Skillings, N. I. Wood, Z. Zheng

**Affiliations:** Department of Physiology, Development and Neuroscience, University of Cambridge, Downing Street, Cambridge, CB2 3DY United Kingdom

## Abstract

Antagonist pleiotropy, where a gene exerts a beneficial effect at early stages and a deleterious effect later on in an animal’s life, may explain the evolutionary persistence of devastating genetic diseases such as Huntington’s disease (HD). To date, however, there is little direct experimental evidence to support this theory. Here, we studied a transgenic mouse carrying the HD mutation with a repeat of 50 CAGs (R6/2_50) that is within the pathological range of repeats causing adult-onset disease in humans. R6/2_50 mice develop characteristic HD brain aggregate pathology, with aggregates appearing predominantly in the striatum and cortex. However, they show few signs of disease in their lifetime. On the contrary, R6/2_50 mice appear to benefit from carrying the mutation. They have extended lifespans compared to wildtype (WT) mice, and male mice show enhanced fecundity. Furthermore, R6/2_50 mice outperform WT mice on the rotarod and show equal or better performance in the two choice discrimination task than WT mice. This novel mouse line provides direct experimental evidence that, although the HD mutation causes a fatal neurodegenerative disorder, there may be premorbid benefits of carrying the mutation.

## Introduction

Huntington’s disease (HD) is a progressive genetic neurodegenerative disorder caused by the unstable expansion of a CAG repeat in the HD gene (*HTT*)^1,2^. It is characterized by a complex set of symptoms that include motor dysfunction, cognitive decline and psychiatric disorder. By end stage of the disease, total brain weight is reduced by 10–20%. This is mainly due to degeneration and atrophy of neurons in the caudate nucleus, putamen and neocortex. There is an inverse relationship between age-at-onset and CAG repeat length, and patients carrying longer alleles typically present as juveniles. In most (>90%) patients, the length of the repeat in the mutant allele causing adult-onset disease is 40–50 CAGs. Understanding why HD is an adult onset disorder is a key question in the field. It is also not clear why the HD gene mutation persists in the population when it causes such a devastating illness.

Pleiotropy is defined as the production by a single gene of two or more apparently unrelated phenotypic effects. Antagonistic pleiotropy is when at least one of these traits is beneficial and at least one is detrimental to the fitness of the organism^3^. The idea that antagonistic pleiotropy plays a role in HD was first suggested by Roger Albin in 1988^4^, although HD had been identified as a possible pleiotropic disease many years earlier by Peter Medawar^5^. In his paper, Albin described apocryphal evidence and also reviewed epidemiological data suggesting that HD patients are more fecund than non-gene carriers^4^. There is both direct and indirect evidence to support this idea^6–8^. More recently, the idea of antagonistic pleiotropy in HD re-emerged, with new evidence showing that there is a reduced incidence of cancer in HD patients^9,10^ and that small interfering (si) RNAs based on the HD gene are toxic to cancer cells^11^. If *HTT* is an example of a gene showing antagonistic pleiotropy, then this may explain, at least in part, why HD persists in the population despite its eventual devastating effects on the individual.

For a gene to be antagonistically pleiotropic it must be selected for beneficial effects early in life; the ‘unselected’ deleterious effects appear post-reproductively. In HD, both improved reproductive fitness and a reduced risk of early onset cancer could be benefits of carrying the mutation. While the mechanisms for improved reproductive fitness are not known, it has been suggested that the reduced cancer risk may be associated with increased activity of the tumour suppressor protein p53^12^. P53 has a role in inducing apoptosis and a higher level of p53 may also protect from tumour growth and cancer development in general.

It would help greatly in understanding possible early benefits of the HD mutation if we knew the role played by the protein product huntingtin (Htt). Htt interacts with many binding partners and has been implicated in many intracellular processes, yet its function remains unknown^13^. It is clear that Htt is essential in development, since deleting both copies of the gene cause early embryonic lethality^14^. By contrast, Htt does not appear to be essential in the adult, since heterozygous knockout mice appear to be normal, as do humans carrying a single copy of the gene^2^. In fact, the role of Htt appears to change with age, since inducible knockout in young mice causes acute pancreatitis, but has no deleterious effect in the adult^15^, and patients with compound heterozygous variants of *HTT* presented with a complex neurodevelopmental disorder that is not HD^16^. If there is an evolutionary advantage to carrying a mutation in Htt, then better understanding of this early role is essential. For example, knowing when a mutation is advantageous may inform us about the timing of disease onset. It is possible that the disease starts only when the balance between the deleterious phenotypic effects shifts to outweigh any potential beneficial effects of a particular mutation. Not only that, but if there are beneficial effects of the HD gene before the onset of disease, this will become an important consideration in deciding when to start treating a patient, particularly with gene therapies that involve permanent knock-down of mutant Htt.

HD is one of the few diseases in which antagonistic pleiotropy can be tested experimentally, because of the well-characterized mouse models available for study. Numerous HD mice models exist that have been used to model every aspect of HD, including brain pathology, behavioral deterioration, CAG repeat instability, gene expression changes, circadian rhythm, and sleep abnormalities^17,18^. Despite their unquestionable value, however, one caveat to these studies is that all of these HD mice carry CAG repeat expansions much longer that those seen in HD patients. Most carry repeats of 110–250 CAGs, and there are no HD mouse models yet characterised that carry a CAG repeat within the typical human range. The shortest repeat length mice shown to cause a behavioral phenotype is 80 CAG repeats in a N171 fragment of HD^19^ and in a ‘knock-in’ mouse^20^. This is at the upper extreme of somatic CAG repeat length seen in juvenile-onset HD patients. In order to test antagonistic pleiotropic effects of the mutation, the CAG repeat length of the mutant allele should be within the range causing disease in humans.

In this study, we describe a novel line of R6/2 mice carrying 50 CAG repeats, which is in the range of repeat lengths causing adult onset HD. By 3 years of age, this mouse has extensive HD-relevant brain pathology in the form of aggregates of mutant Htt. The morphology and distribution of the aggregates is similar to that of aggregate pathology in HD patients, with abundant aggregates in striatum and cortex but few in hippocampus or cerebellum. Remarkably, R6/2_50 mice show no deleterious behavioral effects of the mutation. Rather, they seem to have improved fitness, with increased fecundity, survival and stamina. These findings are consistent with the mutant HD gene exhibiting antagonistic pleiotropy.

## Results

### Origin of the R6/2_50 CAG repeat line of mice

**Figure 1:**
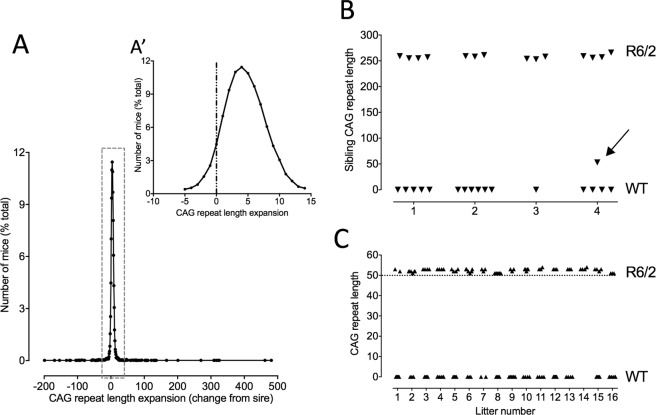
Inheritance of CAG repeat lengths in the parent R6/2 mouse line (R6/2_250) and the offspring of the CAG_50 line founder mouse. The distribution of CAG repeat lengths in the 250 CAG repeat colony (**A**) shows a tendency for a small expansion (A′) compared with the CAG repeat length of the sire. The CAG repeat lengths of all of the TG siblings (triangles in **B**) of the R6/2_50 founder (arrow in **B**) were similar to that of the sire. All of the TG offspring of the founder mouse (N = 16 litters) carried a CAG repeat of ~52. (**C**) Triangles in (**B** and **C**) represent individual mice in separate litters.

The CAG repeat in the exon 1 fragment of the Htt gene in R6/2 mice is inherently unstable (for references, see^2,21^). In our colony, CAG repeat instability in the germline is an expansion of the order of 1–3 CAGs per generation. Occasionally, however, we see a larger expansion. Selectively breeding mice with such expansions has allowed us to establish an allelic series comprising multiple lines of R6/2 mice with different CAG repeats lengths between 110 and >450. For our behavioral studies we typically breed from mice with 250 CAG repeats. Most R6/2 250 mice in our colony inherited a CAG repeat that is similar to (95%) or longer (5%) than their sire (Fig. 1A). Contractions in the germline from 250 CAG are rare. In fact, we have only seen 8 contractions larger than 3 CAGs in the 20 years we have been breeding R6/2 mice.

In 2009, we identified a single mouse carrying 50 CAG repeats. This mouse was the offspring of an R6/2 male mouse carrying 251 CAG repeats and a normal female F1 C57Bl6J/CBA (Fig. 1B). The littermates of this mouse, and all other offspring of the sire carried average CAG repeat sizes of 257 ± 1 (N = 15). We bred from this mouse to establish a colony of 50 CAG repeat mice. We have now bred this line of mice for >20 generations. The mutation is stably transmitted and stable in transmission size. The repeat is pure CAG with no interruptions. The transgenic offspring of this male have a mean CAG repeat of 52.4 ± 0.2 (N = 1072). [Note that because the founder carried 50 CAG repeats, we call this line R6/2_50].

### Greater fecundity in R6/2_50 mice compared to WT mice

In contrast to R6/2 mice with 250 CAG repeats (where females are infertile and males have a short reproductive window and are only capable of siring pups between 5–8 weeks of age) both male and female R6/2_50 mice are fertile. Female R6/2_50 mice carry pups successfully to term, and raise them normally. In fact, they are good mothers, and showed little of the cannibalisation that is typically seen in naïve WT dams. The reproductive capability of male R6/2_50 mice is also enhanced, not only compared to the parent line, but also to WT mice. In WT mice, a decline in breeding is typically seen at 9 months-1 year of age (unpublished observations (AJM) and Jackson Laboratory Resource Manual; http://jaxmice.jax.org/manual/breeding_strategies_manual.pdf). By contrast, R6/2_50 mice are capable of breeding for at least 2 years. Indeed, we have set male breeders up at 2.5 years that have reproduced successfully. The R6/2_50 founder mouse bred for ~2 years (98 weeks), siring 16 consecutive litters from 4 sequential WT breeding females. The last litter sired by that mouse (that survived to 2.5 years) was born 2 weeks after he died. The litter size remained stable (9 ± 1) and the repeat size number passed to offspring did not change significantly over this period (Fig. 1C). We studied the enhanced breeding capabilities systematically with 4 other R6/2_50 mice, replacing the normal female breeder mouse when they stopped breeding. All 4 R6/2_50 male mice continued breeding beyond 2 years.

### General health in R6/2_50 mice and WT mice

**Figure 2:**
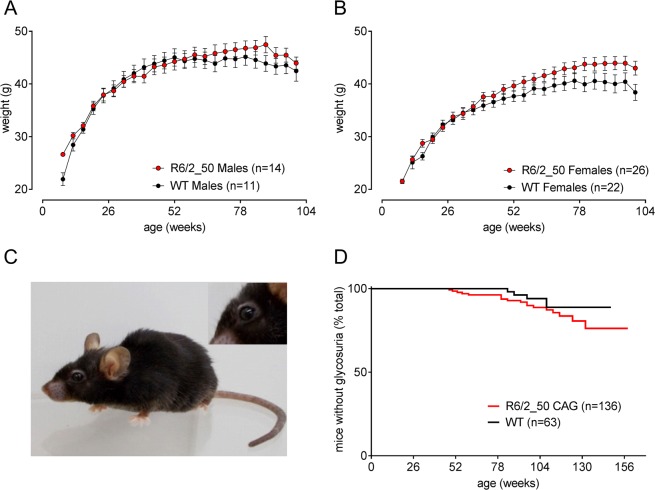
General health of R6/2_50 transgenic mice is better than WT littermates. Mean (±SEM) body weight of male (**A**) and female (**B**) mice show the growth and maintenance of body weight R6/2_50 mice (red symbols) compared to their WT littermates (black symbols). Since the mean body weight, and variance changed when any animals died, the data shown in Fig. 2 are taken from a subgroup of mice within this group that survived for 2 years. The photograph in (**C**) shows an R6/2_50 CAG repeat mouse aged 3 years. The characteristic ptosis is absent, although the fur is greying. Age-related glycosuria was seen in both WT and R6/2_50 mice (P > 0.05).

R6/2_50 mice are physically robust. They show normal growth and do not lose body condition up to 2 years of age (Fig. 2A,B). Not even the oldest R6/2_50 mice (>3 years) show the characteristic ‘R6/2 phenotype’ (cachexia, pronounced lordokyphosis, ptosis). Although they show signs of aging such as greying fur and mild lordokyphosis, they are active with bright wide-open eyes and glossy coats (Fig. 2C).

Glycosuria is observed in R6/2 mice with CAG repeat lengths of 110–250^22,23^. Aged WT mice also show age-related glycosuria. Some, but not all, WT and R6/2_50 mice that survived for 2 years develop glycosuria, but there was no difference between the genotypes (Fig. 2D).

### Improved survival of R6/2_50 compared to WT mice

R6/2_50 mice can live longer than their WT littermates. Soon after we isolated this line, we conducted a small survival study using F2 R6/2_50 mice and their age-matched littermates. Group sizes were not matched because at the start of this study we expected the R6/2_50 mice (N = 68) to die before the WT mice (N = 15). Unexpectedly, when all of the WT mice had died there were still 10 R6/2_50 mice alive. Of the first 50% of mice to die, significantly more WT than R6/2_50 mice died (p < 0.001, χ^2^ 11.6, hazard ratio 3.5). Of the second 50% to die, there was a trend for improved survival in R6/2_50 mice (p = 0.06, χ^2^ 3.4, hazard ratio 2.0). Of the WT mice, 10/15 and 38/68 R6/2 mice survived for 2 years. Only 1 WT mouse (6%) and 16 (24%) R6/2 mice survived for more than 2.5 years. The 4 oldest mice R6/2_50 mice survived for ~3 years. The 6 oldest WT mice that died naturally over the age of 2 years died significantly earlier than the 6 oldest R6/2_50 mice (1023 ± 3 d (~146 w) vs. 1066 ± 12 d (~152 w) respectively, p < 0.01, χ^2^ 9.4, hazard ratio 3.6).

### Improved survival of Hdh 50 CAG repeat mice compared to WT mice

We studied survival in a full length ‘knockin’ line of mice that carries a 50 CAG repeat mutation (Hdh 50; made by Dr Peter Detloff, University of Alabama at Birmingham (Suppl. Fig. 1). Hdh 50 mice also showed a significant improvement in survival compared to the WT littermates, with the second half of the Hdh 50 cohort dying significantly later (Log rank test; p < 0.05, χ^2^ 5.8, hazard ratio 2.0).

### R6/2_50 mice show enhanced motor performance on the rotarod

**Figure 3:**
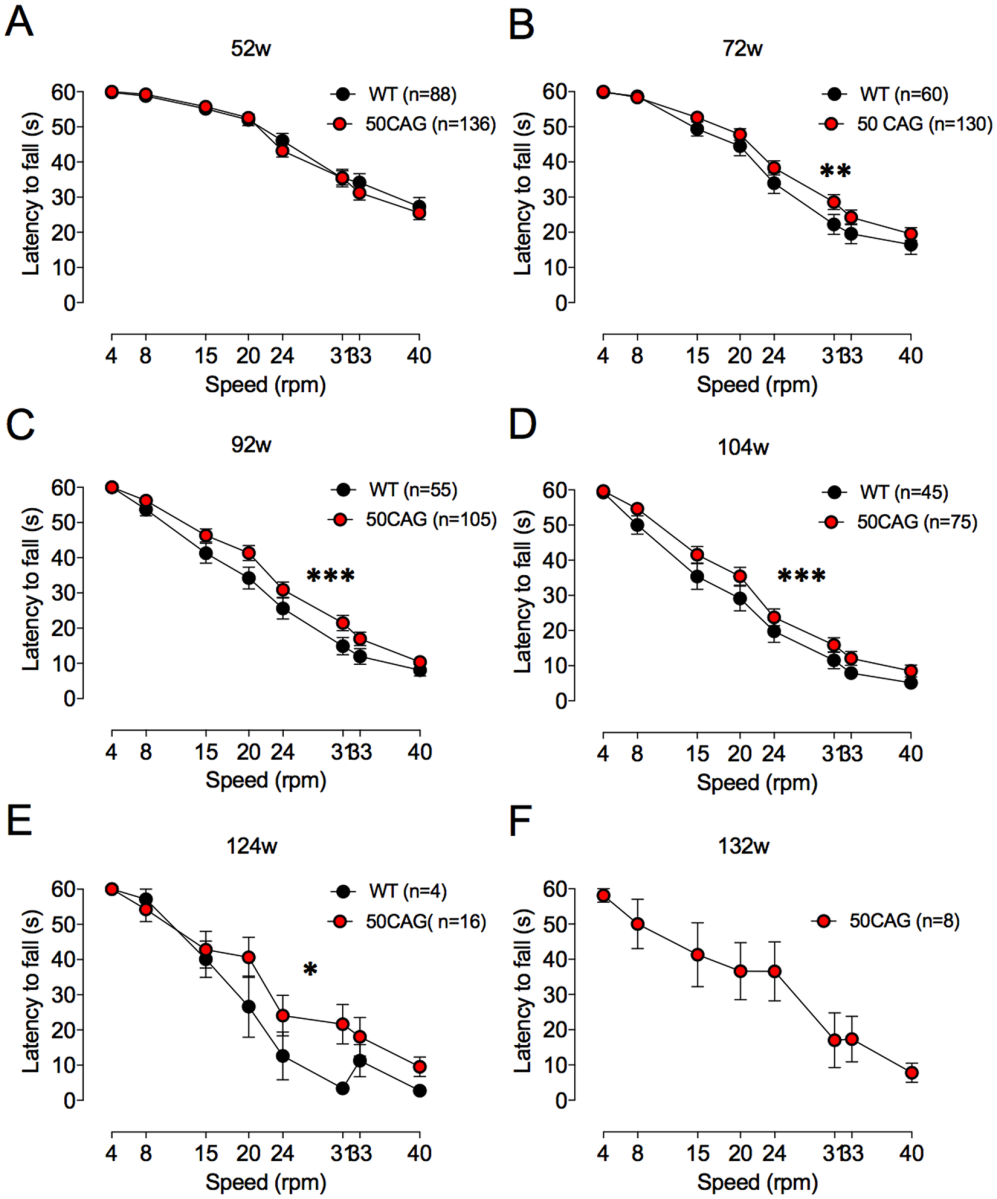
Performance of variable speed rotarod is better in R6/2_50 than WT mice. Rotarod performance is shown at 52 (**A**), 72 (**B**), 92 (**C**), 104 (**D**), 124 (**E**) and 132 (**F**) weeks of age in WT (black symbols) and R6/2_50 (red symbols) mice. At 132 w, only R6/2_50 mice were able to perform on the rotarod. Data points show mean ± SEM. *P < 0.04, **P < 0.002, ***P < 0.001.

Up to a year of age, there was no difference in performance between WT and R6/2_50 mice on fixed speed rotarod (data not shown). By 72 weeks, however, performance of R6/2_50 mice was significantly better than that of WT mice (Fig. 3). From then on, there was a main effect of genotype that persisted until 104 weeks (at 72 weeks, F_(1,1504)_ = 94.60, p < 0.002; 92 weeks F_(1,1264)_ = 16.81, p < 0.001; 104 weeks F_(1,944)_ = 13.58, p < 0.001; 124 weeks F_(1,144)_ = 4.27, p < 0.04). By week 128, all of the WT mice were dead. Week 132 was the last week that surviving 50 CAG repeat mice were tested on the rotarod.

We tested some mice on the accelerating rotarod at two years of age. Not all mice were included, because they could not stay on the rotarod for more than 1 minute. Of the mice that stayed on the rotarod for more than 1 minute (5/10 WT and 9/38 R6/2; p = 0.2), the mean time the mice stayed on the rotarod (shown here as distance that the mice walked) was similar (4.0 ± 1.0 m (N = 5 WT) vs. 5.6 ± 0.8 m (N = 29 R6/2_50). The range of the distances walked by the mice was, however, much greater for R6/2_50 than WT mice. The furthest a WT mouse walked was 6.1 m; the furthest an R6/2_50 mouse walked was 16.9 m. One quarter (8/29) of R6/2_50 mice walked more than 8 m (twice the mean distance walked by the WT mice).

### Improved reaction time in female and decreased performance in male R6/2_50 mice during two-choice discrimination tasks in the touchscreen

**Figure 4:**
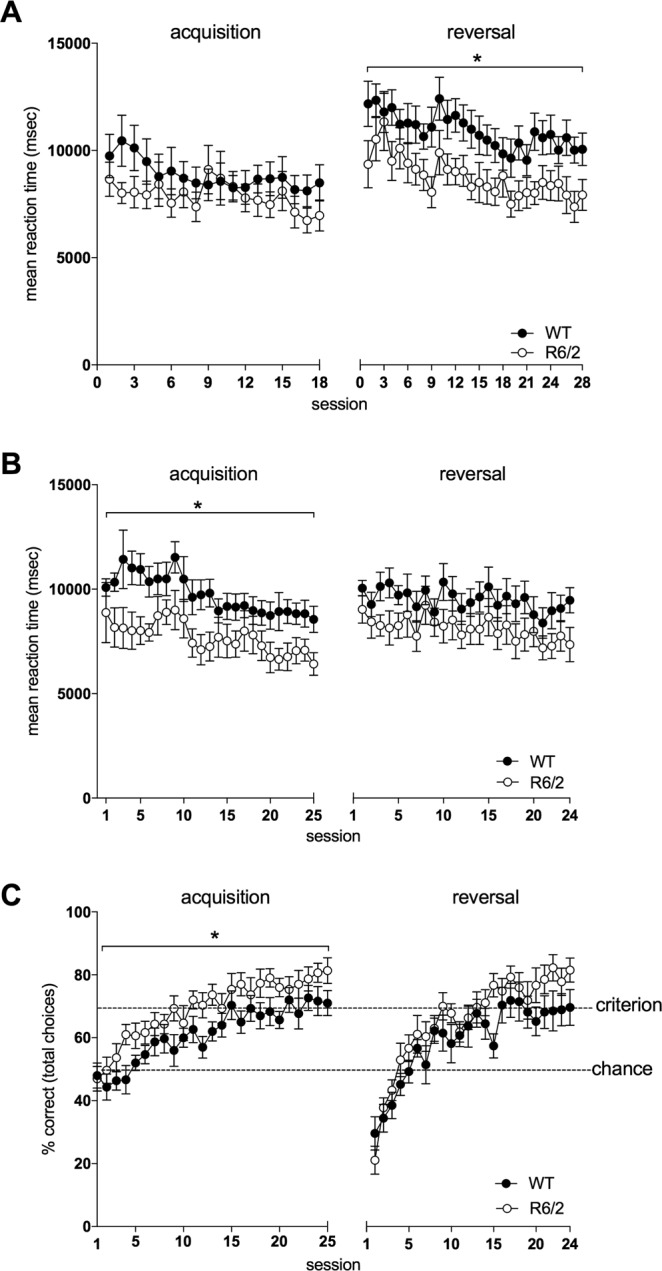
Reaction time and performance of female R6/2_50 mice during cognitive testing in the touchscreen. Data show the mean reaction times (as the time taken to respond to the first presentation of a stimulus pair) during acquisition and reversal during testing Phase 2 (**A**) and 3 (**B**), and performance (percentage of correct choices made) at acquisition and reversal during testing Phase 3 (**C**). Data points show mean ± SEM. For all panels, filled circles (**·**) represent WT mice and open circles (o) represent R6/2 mice. Dotted lines indicate chance (50%) and criterion (70%) levels. *p < 0.05.

We conducted 5 stages of cognitive testing in WT and R6/2_50 mice between 8 months and 2 years of age (for ages and statistics see Table 1). No overall changes in performance were seen in R6/2_50 mice. Some minor individual differences were seen at specific time points. For example, female R6/2_50 mice had a faster reaction time than WT mice during the reversal testing in Stage 2 (Fig. 4A*;* F_(1,18)_ = 4.96, p < 0.05) and acquisition stage of Stage 3 (Fig. 4B; F_(1,18)_ = 6.23, p < 0.05). Female R6/2_50 mice also performed better than their WT counterparts during the acquisition testing in Stage 3 (Fig. 4C, F_(1,18)_ = 5.35, p < 0.05); for other data see Table 1). In the reversal testing of phase 3, male R6/2_50 mice performance (% correct) was slightly worse in the retention phase of the task than WT mice (Suppl. Fig. 2A; F_(1,12)_ = 6.53, p = 0.021), and at 81 and 104 weeks of age male R6/2_50 mice required a greater number of correction trials or sessions to reach criterion (F_(1,13)_ = 7.04, p < 0.05 and F_(1,11)_ = 5.50, p < 0.05 respectively) and were slower to reach criterion in the reversal of Stage 1 testing (Suppl. Fig. 2B). Overall, however, the differences between genotypes were subtle, with some aspects of the cognitive phenotypes improving and others worsening, but most not being measurably different from normal. The clinical relevance of these differences is not clear, although it should be noted that the touchscreen testing protocol was designed to detect deficits, not improvements, in cognitive performance. The group sizes were calculated from previous studies to be sufficiently well powered to detect deficits in performance. Ceiling effects mean, however, that the study is likely to be underpowered for detecting improvements in performance.

**Table 1:** Summary of statistical analyses of touchscreen data.

Testing Stage	Age (weeks)	Performance (% correct)		Time to complete a session		Reaction time (first presentation)		Correction trials (per session)		Number of sessions to reach criterion	
sex		M	F	M	F	M	F	M	F	M	F
**Stage 1**											
Acquisition 1	8	p = 0.202	p = 0.119	p = 0.095	p = 0.451	p = 0.253	p = 0.593	p = 0.279	p = 0.146	p = 0.566	p = 0.230
Reversal 1	11	p = 0.137	p = 0.149	**p** **<** **0**.**05** **R6/2 slower**	p = 0.464	p = 0.248	p = 0.711	p = 0.141	p = 0.445	p = 0.138	p = 0.174
Retention 1	20	p = 0.450	p = 0.136	p = 0.171	p = 0.154	p = 0.931	p = 0.393	p = 0.398	p = 0.523	p = 0.144	**p** **<** **0**.**05** **R6/2 better**
**Stage 2**											
Acquisition 2	21	p = 0.228	p = 0.452	p = 0.606	p = 0.394	p = 0.629	p = 0.372	p = 0.813	p = 0.422	p = 0.279	p = 0.966
Reversal 2	24	p = 0.201	p = 0.574	p = 0.457	p = 0.154	p = 0.423	**p** **<** **0**.**05** **R6/2 faster**	p = 0.287	p = 0.436	p = 0.876	p = 0.413
**Stage 3**											
Acquisition 3	36	p = 0.572	**p** **<** **0**.**05** **R6/2 better**	p = 0.286	p = 0.051	p = 0.286	**p** **<** **0**.**05** **R6/2 faster**	p = 0.100	p = 0.850	p = 0.338	p = 0.077
Reversal 3	41	p = 0.484	p = 0.176	p = 0.286	p = 0.064	p = 0.244	p = 0.156	p = 0.356	p = 0.604	p = 0.350	p = 0.230
Retention 2	64	**p** **<** **0**.**05** **R6/2 worse**	p = 0.322	p = 0.307	p = 0.445	p = 0.931	p = 0.393	p- = 0.337	p = 0.523	p = 0.304	p = 0.573
**Stage 4**											
Acquisition 4	81	p = 0.073	p = 0.258	p = 0.083	p = 0.705	p = 0.574	p = 0.199	**p** **<** **0**.**05** **R6/2 worse**	p = 0.124	p = 0.312	p- = 0.563
**Stage 5**											
Acquisition 5	104	p = 0.068	p = 0.230	p = 0.281	p = 0.712	p = 0.544	p = 0.678	p = 0.069	p = 0.293	**p** **<** **0**.**05** **R6/2 worse**	p = 0.293

### HD-like pathology in striatum and cortex but not hippocampus of R6/2_50 mice

**Figure 5:**
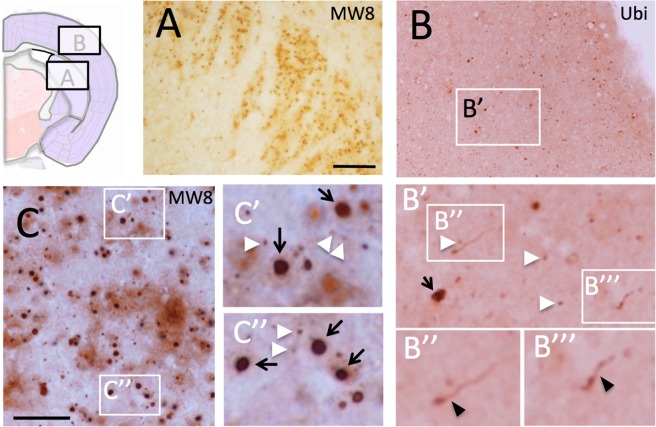
Aggregate pathology is prominent in striatum and cortex in R6/2_50 mice. The photomicrographs show immunoreactivity for aggregated Htt (MW8) in the striatum (**A**,**C**) and ubiquitin in the cortex (**B**). The cartoon indicates the location of the striatal and cortical sections from coronal sections of brain. The white arrowheads indicate examples of classic nuclear inclusions; the black arrows indicate examples of large extranuclear aggregates; the black arrowheads indicate ubiquitin-positive material in neuritic processes. The bars in A = 200 µm for (**A**,**B**) and 20 µm for (**C**). For other histology, see Suppl. Figs 1–7.

Progressive mutant Htt aggregate pathology (as revealed using MW8 and ubiquitin immunohistochemistry) is seen in the brains of R6/2_50 mice. At 1 year of age, occasional small (<2 µm diameter) aggregates of mutant Htt are present in cortex and striatum of R6/2_50 mice. By 2 years of age, there is an increase in the number of cells containing aggregates, in both cortex and striatum. By 2.5 years, cortical and striatal aggregates are abundant, as are aggregates in some nuclei in the thalamus. We had 4 mice survive to 3 years of age. The brain size of all of these mice was normal, and their macroscopic morphology was also normal. Nevertheless, there were numerous Htt-positive aggregates seen in these brains, particularly in striatum and cortex (Fig. 5 and Suppl. Figs 3–6). Neuropil aggregates were very common, with multiple shapes and forms of immune-positive material that included neuropil threads, ‘beads-on-strings’, granular aggregates and diffuse cytoplasmic immunoreactivity. Every form of aggregate that has been identified in HD brain^24,25^ was seen in the R6/2_50 mouse brain. Although the aggregate density and morphology varied from mouse to mouse, the pattern of aggregate distribution was similar. Aggregates in three of the mice were sparser and smaller than those present in the fourth mouse. In mouse 4, the pathology seemed more advanced than that of the other mice, with more numerous and larger aggregates. We have therefore used this mouse to illustrate the end stage pathology in this line of mice.

In mouse 4, MW8-positive aggregates were found primarily in the striatum (Fig. 5A,C and Suppl. Fig. 3) and cortex (Fig. 5B and Suppl. Figs 4–6). Aggregates were also found in the thalamus, substantia nigra and molecular layer of the cerebellum (data not shown), but none were found in principal neurons of the hippocampus (Suppl. Fig. 7). Aggregates varied in size and shape, and displayed all of the morphologies described for aggregates in HD brain^24,25^ (Fig. 5 and Suppl. Figs 3–6). These included the characteristic neuronal nuclear inclusions (NIIs) as well as perinuclear and neuritic aggregates typically seen in HD mice^26^. Neurons in the dorso-lateral striatum showed a higher density of NIIs than any other part of the brain. In cortex, NIIs were found predominantly in neuronal nuclei in layer 5/6 but also occasionally in layer 2. Extranuclear aggregates were found in all cortical layers, with many small inclusions in linear format, consistent with their presence in neurites. In the hippocampus, there were no aggregates seen in principle neurons of Cornu Ammonis (CA), or in the dentate gyrus (Suppl. Fig. 7). This is in marked contrast to the pathology typically seen in mouse models with longer CAG repeats, where aggregates appear first in CA1^26,27^ and are eventually found in all classes of hippocampal neurons, including granule neurons of the dentate gyrus. Interestingly, as seen in HD patients^24^, significant Htt immunoreactivity was seen in white matter, with aggregates present in corpus callosum, fibre bundles in the striatum, and white matter in the cerebellum (Suppl. Fig. 8).

## Discussion

We describe a novel line of HD mice carrying a mutation with a 50 CAG repeat expansion that is in the range causing adult–onset HD. This line of mice has two striking and unusual features. First, the *post mortem* aggregate pathology is more similar to the pattern seen in HD than of any line of mice reported hitherto, with aggregates appearing predominantly in the striatum and cortex, but not in hippocampus or cerebellum. Although some mice, e.g. the Q140 knockin^28^, and the Hdh 150 knockin mouse^29^, show first presentation of inclusions predominantly in the striatum, both of these lines eventually develop aggregates in all brain regions, including hippocampus^28^ and cerebellum (unpublished observations, AJM). Second, despite the eventual widespread HD-like pathology, R6/2_50 mice do not show pronounced behavioral abnormalities. On the contrary, they appear to benefit from carrying the mutation, since they are more robust than their WT littermates (as judged by their superior performance in locomotor tasks, reproductive fitness, and survival). A second line of HD mice with a CAG repeat mutation of 50 carried as a ‘knockin’ also shows improved survival compared to WT mice. Together these data support the idea that the mutant HD allele shows antagonistic pleiotropy, with beneficial effects on multiple domains in adult mice, despite the eventual development of HD-like brain pathology that is the harbinger of full-blown disease.

The R6/2_50 line of mice shows the desired regional specificity of CAG-related pathology that has been so lacking in HD mouse models to date. Thus, it appears that differential regional distribution of aggregates depends more on the length of the CAG repeat expansion than its genetic context, since R6/2_50 mice carry the CAG repeat expansion in only a fragment of *HTT*. This should resolve one of the concerns of the field about HD models in general, and R6/2 lines in particular. The aggregate brain pathology in R6/2 mice with repeat lengths between 110–250 does not start in the striatum or cortex, the regions that degenerate in HD^2^. Rather, aggregates appear first in the hippocampus and then appear rapidly in all other parts of the brain, including the cerebellum^26,27^. This widespread pathology, along with misgivings about the rapid onset of disease in R6/2 mice, triggered a debate focused on the appropriateness of fragment models that has continued for many years, despite *prima facie* evidence that fragment models recapitulate many of the genetic changes seen in HD^30^, and that mice carrying the HD mutation in a more ‘relevant’ genetic context (e.g. full length knockin Hdh Q150, and BAC HD lines) show a widespread pattern of aggregate brain pathology^29,31^. The restricted HD-like cortico-striatal histopathology in the R6/2_50 mice suggests that the widespread pathology in most HD models is related to the exaggerated CAG repeat size, rather than the nature of the transgene (knockin vs. fragment). Notably, the HD-relevant pathology of the R/2_50 mouse offers a unique opportunity to study the regional specificity of early brain pathology in HD, an understanding of which has been particularly elusive.

Whilst there are at least two other mouse models that carry short repeats (~50CAG), a full length cDNA transgenic mouse^32^ and a knockin mouse^33^, the transgenic mouse line had extensive aggregate pathology in multiple regions, including cortex, striatum and hippocampus and the mice died at around 25 weeks^32^. The knockin mouse showed appeared normal up to 6 months, but was not characterised past that age. Two lines of transgenic (tg) HD rat have also been generated, that carry a CAG repeat of 51 (SPRDTgHD^34^ and F344tgHD^35^). Both HD rat lines show Htt-positive aggregate pathology by 21 months, particularly those regions associated with regulation of emotion (nucleus accumbens, amygdala), although up to 21 months neither strain show strong cortical staining and both show significant hippocampal staining^34,35^. Clearly, differences in transgene construct, promoter and expression levels and species differences, as well as different antibodies used for visualisation of brain pathology make direct comparisons of these lines of HD rodent models with the R6/2_50 mouse inadvisable. As with the R6/2_50 mice, the tgHD rat cognitive phenotype evolution is complex, with both improved and worsening phenotypes being measurable compared to WT rats. It is nevertheless intriguing to note that at an early age, both lines of tgHD rats are more social and less anxious than WT rats^35,36^ and the performance of young SPRDTgHD rats on the rotarod is better than that of the WT controls^36^. SPRDTgHD rats also showed better learning and reacquisition of discriminative fear conditioning^37^.

We show direct evidence that antagonistic pleiotropy may play a role in the phenotypic development of the R6/2_50 repeat mice. A retrospective review of the literature reveals, however, that there are numerous examples of potentially beneficial effects of the CAG repeat mutation in HD mouse models, even in lines that have rapid disease onset. Most notable are studies whereby HD mice are shown to be resistant to the effect of exogenous neurotoxins. For example, despite the rapidly progressing deleterious phenotype seen in mice carrying repeat lengths of 140–250 CAGs, R6/2 mice are resistant to the effects of several neurotoxins including quinolinic acid^38,39^, kainic acid^40^, 3-nitropropionic acid^41^, N-methyl-d-aspartate (NMDA) and malonate^42,43^ as well as to ischaemic insult^44^. Mice from a related line (R6/1) are resistant to global cerebral ischaemia^45^, and N-171-82Q mice^46^ and YAC mice carrying 140 CAGs^47,48^ show resistance to excitotoxicity caused by NMDA and/or quinolinic acid. FVB/N mice, which are highly vulnerable to excitotoxicity, also become resistant to quinolinic acid-induced striatal neurodegeneration with age, when carrying a full length Htt transgene carrying a CAG repeat 140^48^. Finally, R6/2 mice show reduced sensitivity to neurotoxicity induced by dopamine and 6-hydroxy dopamine^49^. Although the mechanisms underlying resistance to exogenous insults remain elusive, they appear to be independent of short-term expression of endogenous neuroprotective proteins^39,45^. A number of groups have tried to link the endogenous neuroprotection in HD mice with particular pathways, e.g. p53^50–52^, but to date, no clear mechanism has emerged. Nevertheless, together these studies show that the presence of a pathological length CAG repeat results in a dramatic resistance to damaging, and in some cases lethal, excitotoxic insults.

As well as resistance to excitotoxins, there are other mutation-related changes in HD mice that might be beneficial. Although body weight loss is a complex marker, both YAC mice and BAC HD mice are heavier than WT mice^53,54^, suggesting that the third copy of the *HTT* gene is beneficial. A heavier body weight is correlated, not only with better survival and delayed onset of disease in HD mice^55,56^ but also with a slower progression of disease in HD patients^57,58^. While it is not clear if deliberately maintaining a good body weight in HD patients can slow the disease, there is no doubt that body weight loss in HD patients typically heralds the beginning of the end-stage of HD. Interestingly, behavioural testing shows that BACHD mice exhibited higher efficiency of reward collection than WT animals, a characteristic that became more pronounced with age^59^. Consistent with this, there is some evidence that premanifest HD gene mutation carriers show enhanced perceptual learning^60^. Finally, early behavioural data from our lab show that R6/2 mice (with repeats of ~140) have superior cognitive performance to that of WT mice at presymptomatic ages (see Fig. 5B,C in ref.^61^). This is interesting, since this line of mice with a CAG repeat of ~140 had a rapidly accelerating phenotype, and at 8 weeks, their reproductive capacity was already severely compromised. Thus, the beneficial effects of the transgene on cognition were dissociated from the reproductive capabilities of these mice.

The core tenet of the antagonistic pleiotropy theory is that the forces of natural selection weaken with age and that the selective value of a gene depends on how it affects the total reproductive capacity. If a fatal disease such as HD happens late in life, its consequences will be completely unimportant to the survival of the population. Indeed, it was only in the 19th century that life expectancy at birth outstripped the age-at-onset of HD. It is notable that not only does HD persist in the population, but it may also increase in frequency. This was predicted by both Shokeir and colleagues^62^ and Rubinsztein *et al*.^63^. In fact, there has been an increase in the prevalence in HD recorded in the UK^64,65^, although it remains to be seen if this is due to genetic factors, or to improved methods of collecting epidemiological data.

Variation in the CAG repeat length within *HTT* seems to exhibit a form of antagonistic pleiotropy that perfectly illustrates that proposed by Medawar. Unfortunately, HD allele carriers do not enjoy better health. Furthermore, although there is some evidence that there is reduced cancer in HD, this is not a hard-and-fast rule, since it appears to depend on the type of cancer involved^66^. Most recently, there is evidence that CAG/CUG repeats may be toxic to cancer cells^11^. This raises the intriguing possibility that CAG repeats are part of a natural defence mechanism against tumours. We did not systematically investigate tumour formation in our mice, although it would clearly be interesting to do so in a future study.

For many years, there has been an interesting debate about the mechanism(s) underlying the persistence and prevalence of the expanded CAG repeat that causes HD^67–72^, and not all of the proposed models require the expanded CAG repeat to be subjected to negative selection. In 2011, however, Carter and Nuygen reviewed the idea that antagonistic pleiotropy is a feature of human genetic diseases, including HD^73^. They argued that rather than being the ‘exception to the rule’, antagonistic pleiotropy might be a fundamental mechanism for the survival of these non-optimal alleles. Most of the diseases Carter and Nuygen considered are recessive human genetic diseases, with heterozygous carriers being protected against (usually) infectious diseases, citing well known evidence showing that heterozygote carriers for sickle cell anaemia and beta-thalassemia are protected against malaria, and that carriers of the mutation for cystic fibrosis have more offspring and may experience increased resistance to cholera and other infectious diseases. They also cite evidence that Tay Sachs disease, a severe neurodegenerative disease caused by a number of different mutations in the HEXA gene, may owe its surprisingly high frequency to benefits provided by protection from tuberculosis during the historical process of urbanization^72–74^. HD was used as the prime example of a neurodegenerative disease that might exhibit antagonistic pleiotropy, based on the increased fertility and decreased risk of cancer. It would be interesting if the HD mutation also protects against infection.

The dynamic nature of the mutant Htt-mediated phenotype means that therapeutic approaches to HD may need to be ‘personalized’ according to the stage and development of the disease. For example, one promising proposed method for treating HD is by silencing gene expression^75–77^. Treatments that significantly silence the expression of an antagonistically pleiotropic allele, however, may lead to a reduction not only in deleterious but also in any beneficial effects. By eliminating a problem, we may also eliminate a benefit, such as endogenous protection against excitotoxicity or tumorigenesis. A better option, if it could be developed, would be to target the expanded CAG repeat itself, rather than the whole gene. In the meantime, we suggest that particular attention needs to be paid to timing and duration of genetically-based treatment of diseases caused by genes that may show antagonistic pleiotropy. Understanding more about the beneficial effects of pleiotropic genes that cause late-onset neurodegenerative disease should be part of the critical pathway towards developing future therapies.

## Materials and Methods

### Mice

This research was regulated under the Animals (Scientific Procedures) Act 1986 Amendment Regulations 2012, and following ethical review by the University of Cambridge Animal Welfare and Ethical Review Body. Mice were taken from a colony of R6/2 mice established in the University of Cambridge, and maintained by backcrossing onto CBA × C57BL6 F1 female mice. Details of the origins of the R6/2_50 CAG repeat mice are reported in the Results.

Genotyping methods and detailed husbandry have been described previously^78^. Briefly, mice were housed in single-sex, mixed genotype groups. Water and food were provided *ad libitum* except for mice undergoing cognitive testing that were placed on a restricted diet for the duration of touchscreen testing periods, such that their body weight did not fall below 85% of their free-feeding weight. Genotyping and CAG repeat length measurements were carried out by Laragen (Los Angeles, CA, USA) and reported as determined by GeneMapper.

Survival was assessed in an early generation of R6/2_50 mice (N = 15 WT and 68 R6/2_50). Death was recorded if mice were either found dead or if they were killed at the humane end point (failure to exhibit a righting reflex or to rouse after gentle stimulation).

Separate cohorts of mice were used for behavioural testing. N = 47 WT and 113 R6/2_50) were trained and tested on the rotarod (Ugo Basile, Varese, Italy). Body weight was measured in another group of aged-matched littermates from the same generation (N = 94 WT and 120 R6/2_50) that did not undergo rotarod or any other behavioral testing. All mice were weighed twice weekly. Four cohorts of 10 mice of each sex and genotype from a later generation were used for cognitive testing (40 mice in total).

Glycosurea was measured randomly in some mice from all groups using Diastix (Bayer, Basel, Switzerland).

### Behavioural testing

#### Rotarod

For rotarod testing, each mouse received 2 training trials of 60 s per day for 4 days at a fixed speed of 24 rpm. For the testing, each mouse was given 2 successive trials at each of 7 different speeds (5, 8, 15, 20, 24, 31, 33 and 40 rpm) for one minute. At each speed, the latency to fall from the rotarod was recorded. The average of both trials at each speed was used for the analysis. In addition, some of these mice were tested once on the accelerating rotarod at 2 years (N = 5 WT and 29 R6/2_50). The rotarod was accelerated from 4–40 rpm over a 10 minute period and latency to fall was recorded. The mean time from three trials (conducted on the same day with an inter-trial interval of 2 hours) was used to calculate distance walked.

#### Two-choice discrimination in the touchscreen

The testing apparatus consisted of operant testing chambers with touchscreens as previously described^79^. Following initial training, there were 5 separate stages of testing (Table 1), using two different pairs of brightness-matched stimuli. A correct choice elicited a food reward (14 mg 5 TUL purified rodent pellets; TestDiet, MO, USA), while an incorrect choice resulted in a ‘time-out’, whereby the house light was extinguished for a period of five seconds. This was followed by a correction procedure, where the mouse was required to repeat the trial until a correct choice was made. All mice completed all stages of testing.

At Stage 1, mice were tested from 8 weeks of age on the acquisition of a two choice discrimination (Marbles S+: Fan S−). After 14 sessions of acquisition testing the stimuli were reversed (Fan S+: Marbles S−). Mice were then tested for a further 24 sessions using the new stimulus-reward contingency (Fan S+: Marbles S−). After a 5 week break from testing, retention was tested over 7 sessions. At Stage 2, mice (now aged 21 weeks) were presented with a new stimulus pair (Wing S+; Nut S−). They were tested for 18 sessions of acquisition learning, after which the stimuli were reversed. Mice were then tested on the reversal phase of the task for 50 sessions. At Stage 3, mice (now aged 36 weeks) were started on a new phase of testing using the original stimulus pair (Marbles S+/Fan S−). They were presented with 25 sessions of acquisition testing followed by a further 24 sessions of reversal testing. Following a 20 week break from testing, the mice were tested on the retention of the reversal S+ (Fan) for 7 days. At Stage 4, the mice (now aged 81 weeks) were retested on the stimulus pair - Marbles S+/Fan S− for 14 sessions. At Stage 5, the mice (now aged 2 years), were tested for a final time using Marbles S+/Fan S−.

Each of the stages of testing was analysed in terms of performance (percentage correct), reaction time (time taken for the mouse to respond to the initial presentation of the stimulus), number of correction trials required to complete a session, and mean number of sessions required to reach criterion.

### Immunocytochemistry

The antibody staining procedure was performed essentially as previously described^27^, using cryosections (30 µm) of fresh frozen brain tissue. Antibodies used were a rabbit polyclonal anti-poly-ubiquitin (1: 2000; Dako, Denmark) and MW8, a mouse monoclonal antibody that recognises the aggregated conformation of mutant Htt (1: 2000; kind gift of the late Paul Patterson, Caltech, CA, USA). Secondary antibodies were horseradish peroxidase (HRP)-conjugated IgG (1: 2000; Vector, Peterborough, UK). Immunoreactivity was visualized using diaminobenzidine (Sigma, Poole, UK). Note that we describe the proteins visualized by MW8 as aggregated ‘mutant Htt’. This is not strictly accurate, since the transgene contains only the first exon of the HD gene. Thus, the major protein species found in inclusions is not full length Htt, but rather is a mutant fragment of Htt with a pathological length polyglutamine repeat.

### Statistical analysis

Statistical analyses were performed using Statsoft Statistica v13 software (Statsoft, Tulsa, Oklahoma, USA). Unless otherwise stated, data from male and female mice were analysed separately, using repeated-measures analysis of variance (ANOVA), with genotype as a factor. Bonferroni’s post-hoc tests were performed when significant group effects were identified. Survival comparison was tested with the Log rank test. Significance levels were set at p < 0.05.

## Supplementary information

Morton et al Supplementary Figures
