# Supplementary material for: Antagonistic pleiotropy in mice carrying a CAG repeat expansion in the range causing Huntington’s disease
Source: Sci Rep. 2019 Jan 10;9:37. doi: 10.1038/s41598-018-37102-8 (PMC6328633; doi:10.1038/s41598-018-37102-8)
Supplement: Supplementary file 1 — Morton et al Supplementary Figures [file 41598_2018_37102_MOESM1_ESM.pdf]

Antagonistic pleiotropy in mice carrying a CAG repeat expansion in the range causing Huntington's disease

A. J. Morton, E. Skillings, N. I. Wood, Z. Zheng

Department of Physiology, Development and Neuroscience, University of Cambridge, Downing Street, Cambridge, CB2 3DY, United Kingdom

Supplementary Figures 1-9

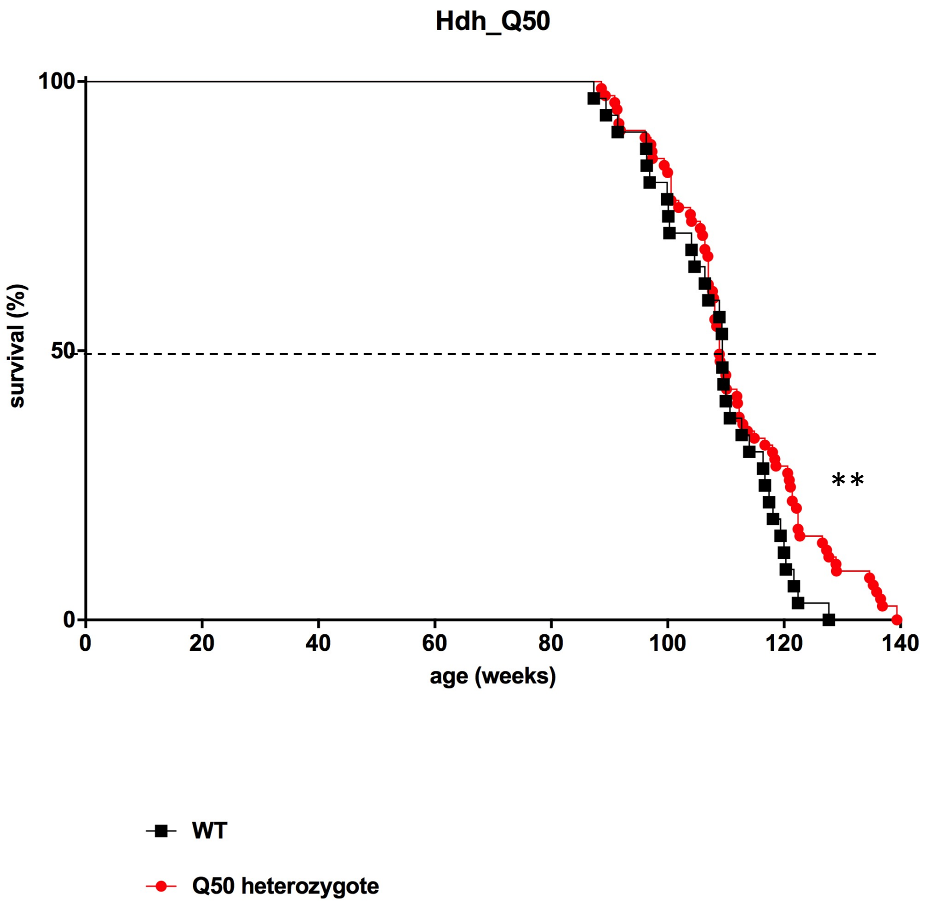

Supplementary Figure 1. Improved survival of Hdh_50 CAG repeat mice.
There was no genotype difference in age-at-death in the first 50% of mice to die, but of the second 50%, Survival of Hdh_50 mice was significantly longer than that of WT littermates (*=P<0.02; Logrank test. N=30 Q50 Het, N=16 WT mice)

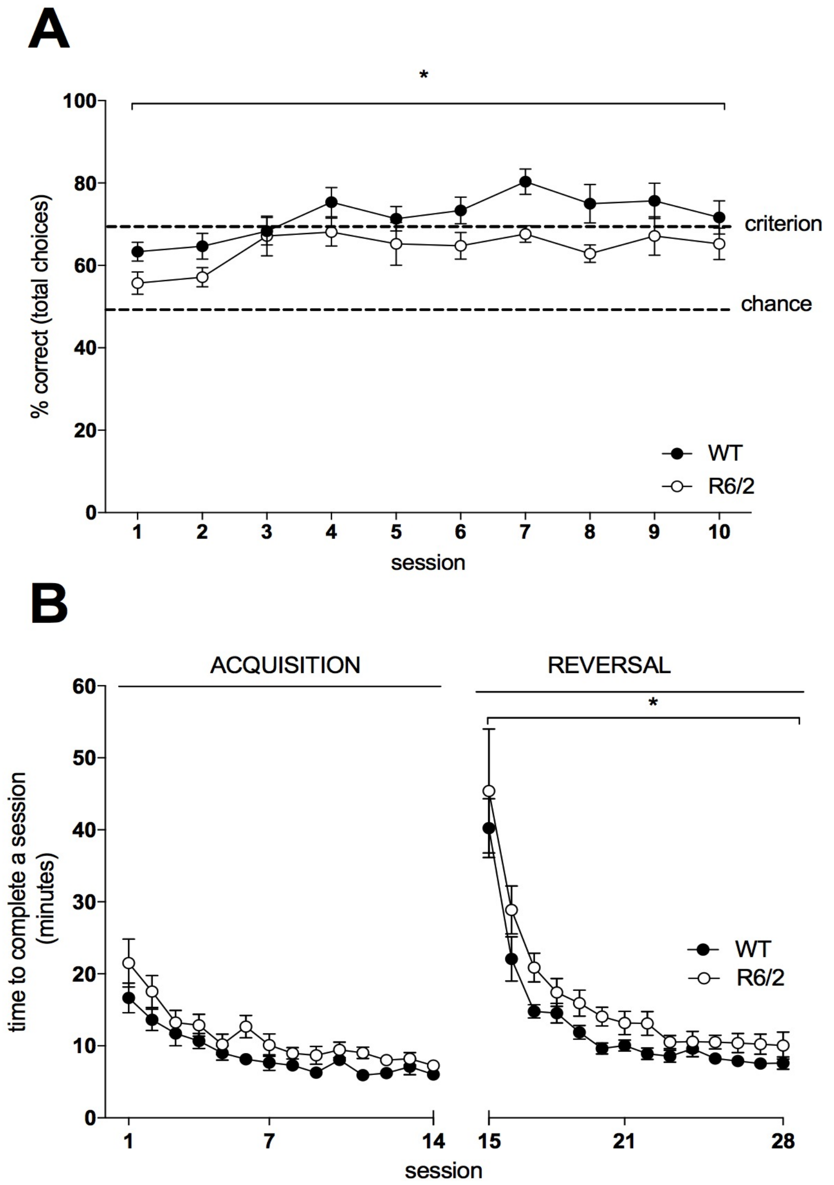

Supplementary Figure 2. Cognitive performance in the touchscreen of male mice is impaired in selected tests. R6/2_50CAG repeat mice show slightly worse performance (percentage of correct choices made) at stage 3 testing (retention 2; A), and take longer to complete a 30 trial session during stage 1 testing (B). No other deficits were seen at any stage or time of testing. Data points are mean ± SEM. For all panels filled circles (•) represent WT mice and open circles/bars (o) represent R6/2 mice. Dotted lines indicate chance (50%) and criterion (70%) levels. * indicates significant difference between WT and R6/2 mice *p<0.05

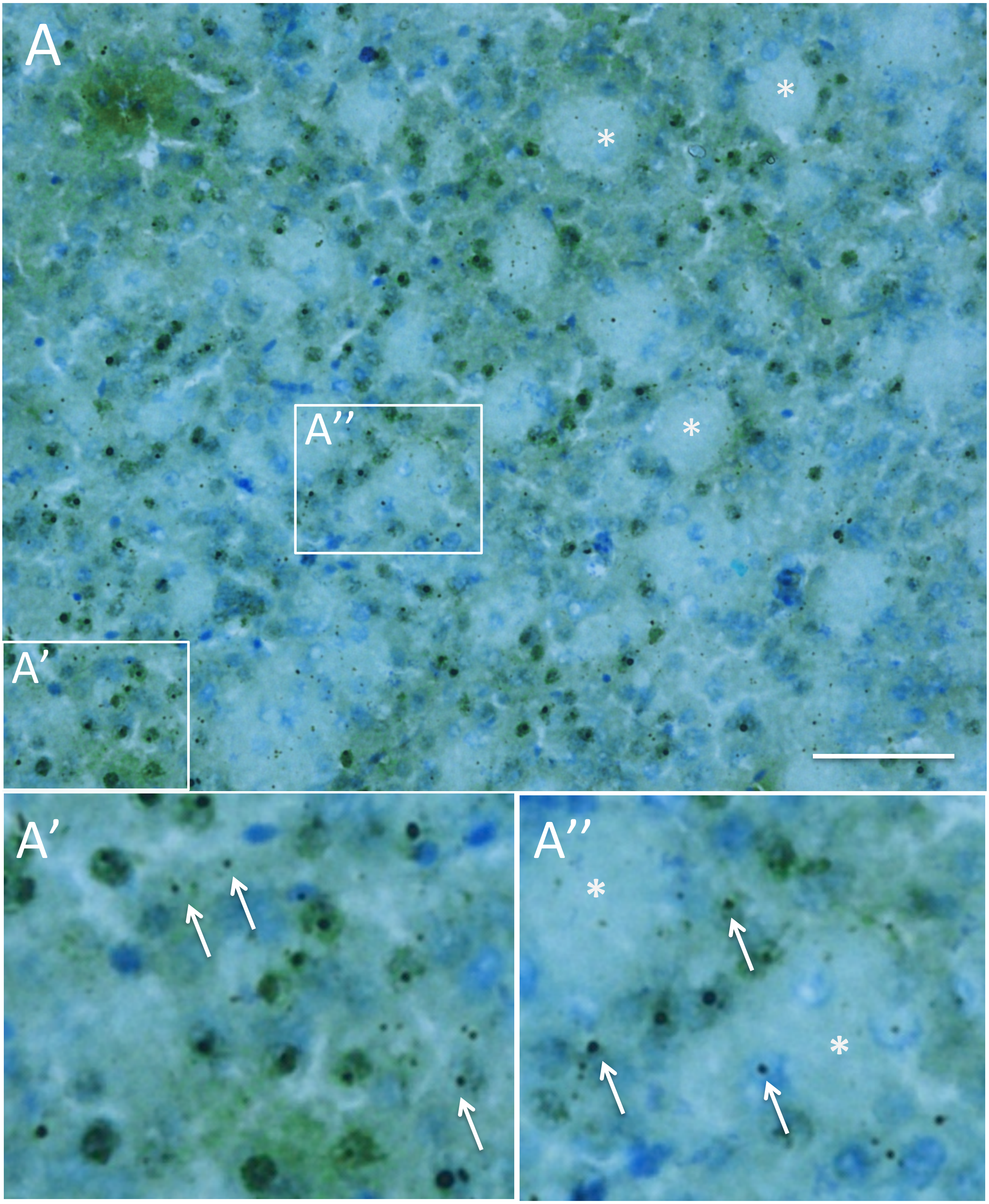

Supplementary Figure 3. Photomicrographs showing extensive aggregate pathology in striatum of an R6/2_50 mouse. The sections have been immunostained with MW8 (brown) and counterstained with methyl blue (blue). * indicates examples of fibre bundles. Size bar in A = 25µm. Examples of neuronal intranuclear inclusions (arrowhead).

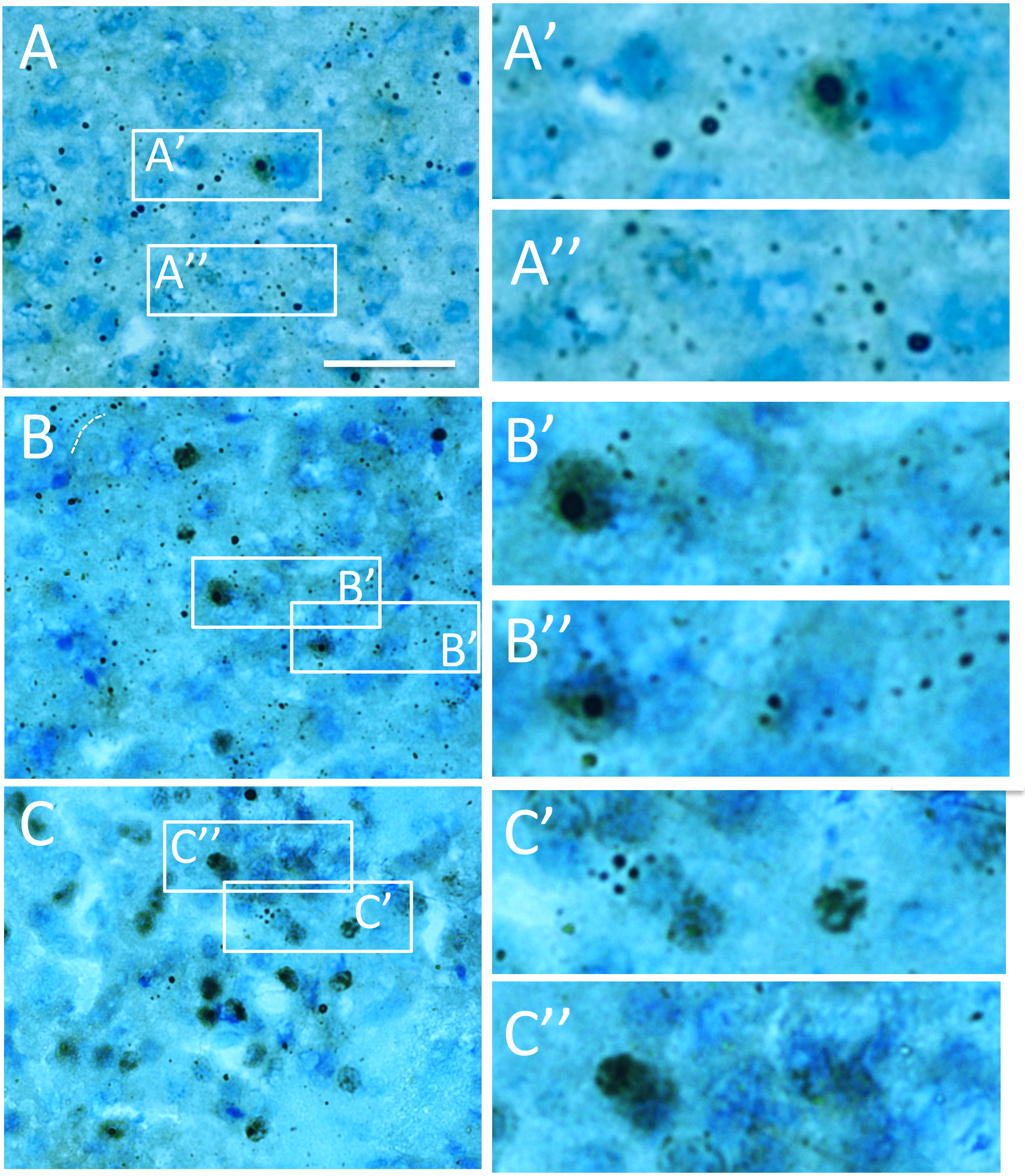

Supplementary Figure 4. Photomicrographs showing aggregate pathology in cortex of an R6/2_50 mouse. The sections have been immunostained with MW8 (brown) and counterstained with methyl blue (blue). Size bar in A = 50µm for A, B and C.

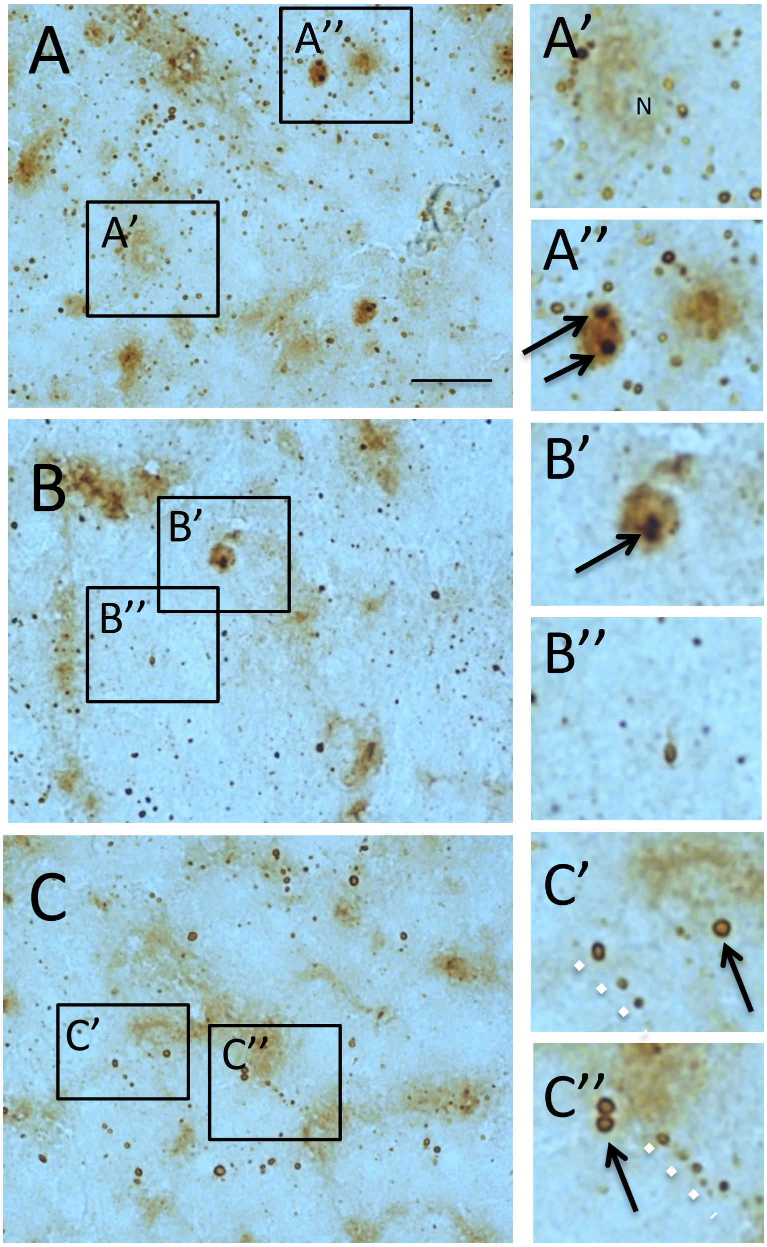

Supplementary Figure 5. Photomicrographs showing that there are multiple morphologies of aggregates in cortex, including extranuclear (A'), intranuclear NNIs (arrows, A'', B', C', C''). Some extranuclear aggregates are organised in linear arrays, consistent with their presence in neurites (white dotted line in C', C''). The sections have been immunostained with MW8 and counterstained with Hoechst 33342 (not visible). Size bar in A = 50μm for A, B and C.

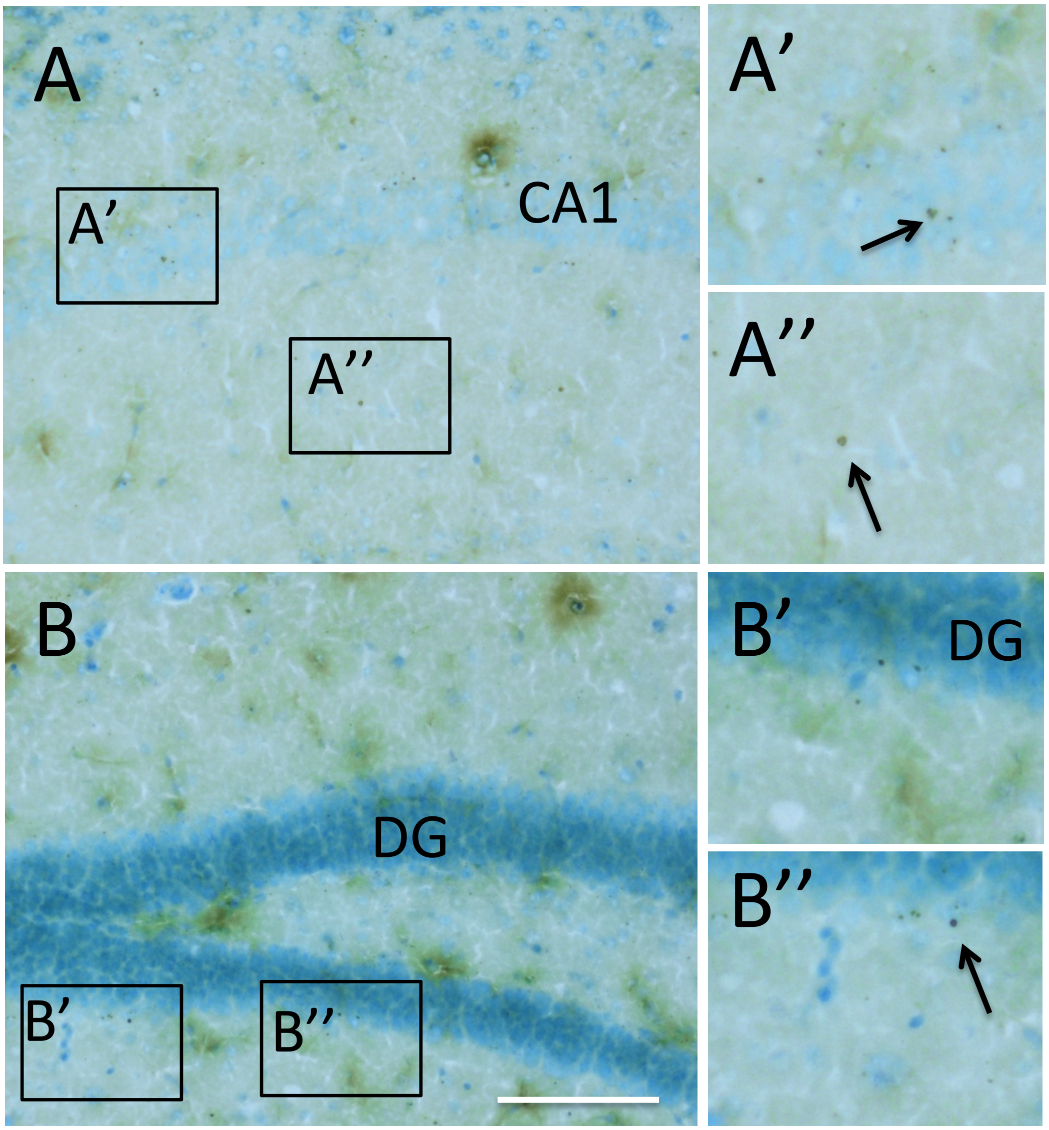

Supplementary Figure 6. **Neuronal nuclear inclusions do not form in principal neurons in the hippocampus.** Photomicrographs show that there is little aggregate pathology in the hippocampus of R6/2_50 CAG repeat mice, even in mouse 4 (shown here) that hds the most pronounced striatal aggregate pathology. There are no visible aggregates in the principal neurons of layers CA1 (A), CA2-4 (not shown) or in the dentate gyrus (DG; B). A few isolated aggregates are visible in the hippocampus (A’, A’’, B’, B’’). The sections have been immunostained with MW8 and counterstained with methyl blue. DG = dentate gyrus; Size bar in B = 200µm.

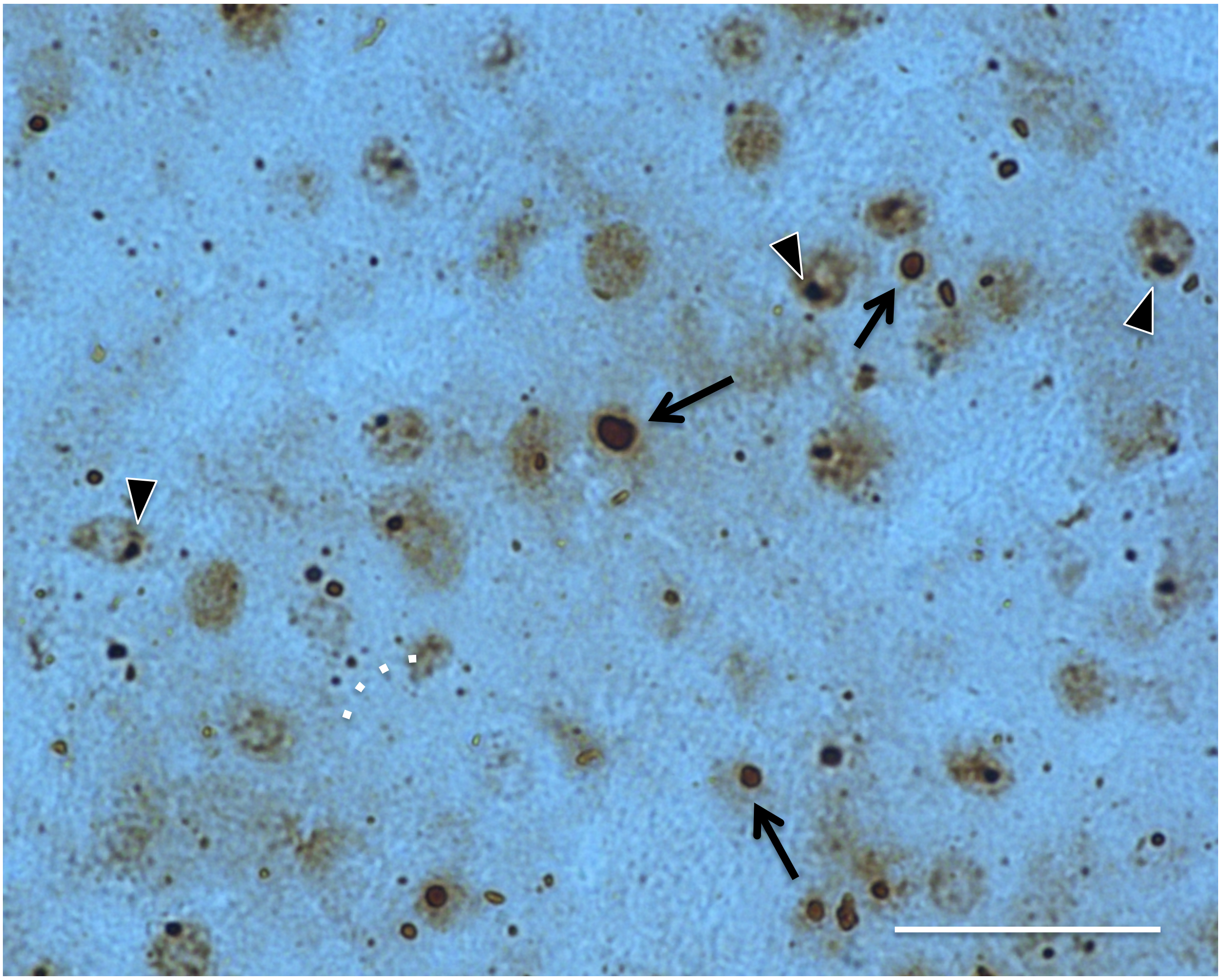

Supplementary Figure 7. **Examples of multiple isoforms of aggregates in R6/2_50 mouse striatum at 3 years of age.** The photomicrograph showing large (black arrows) and small neuronal nuclear inclusions as well as examples neurons with diffuse cytoplasmic staining and extranuclear inclusion (arrowheads). Numerous small cytoplasmic aggregates are present in the striatum. Some extranuclear aggregates are organised in linear arrays, consistent with their presence in neurites (dotted line). The sections have been immunostained with MW8 and counterstained with Hoechst 33342 (not visible). Size bar in A=20μm.

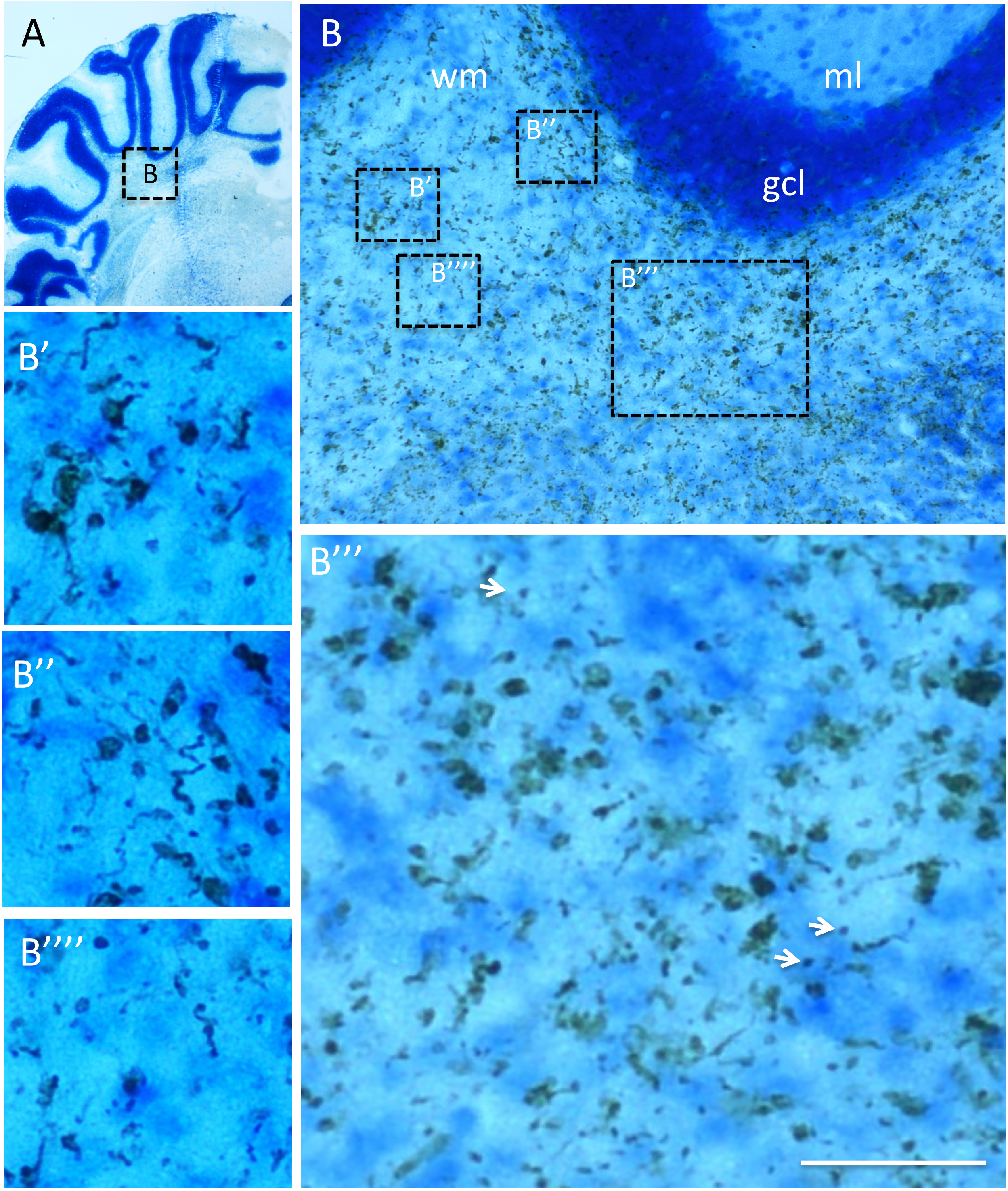

Supplementary Figure 8. White matter aggregates in the cerebellum of an R6/2_50 mouse. Photomicrographs showing extensive aggregate pathology in white matter of R6/2_50 cerebellum. Some aggregates appear to be classic nuclear inclusions (arrowheads in B'''), but most have irregular morphology. Many of the non-nuclear aggregates have a twisted or corkscrew appearance (B'-B''''). The sections have been immunostained with MW8 (brown) and counterstained with methyl blue (blue). gcl = granule cell layer, wm = white matter, ml = molecular layer. Size bar in B'''=10µm.

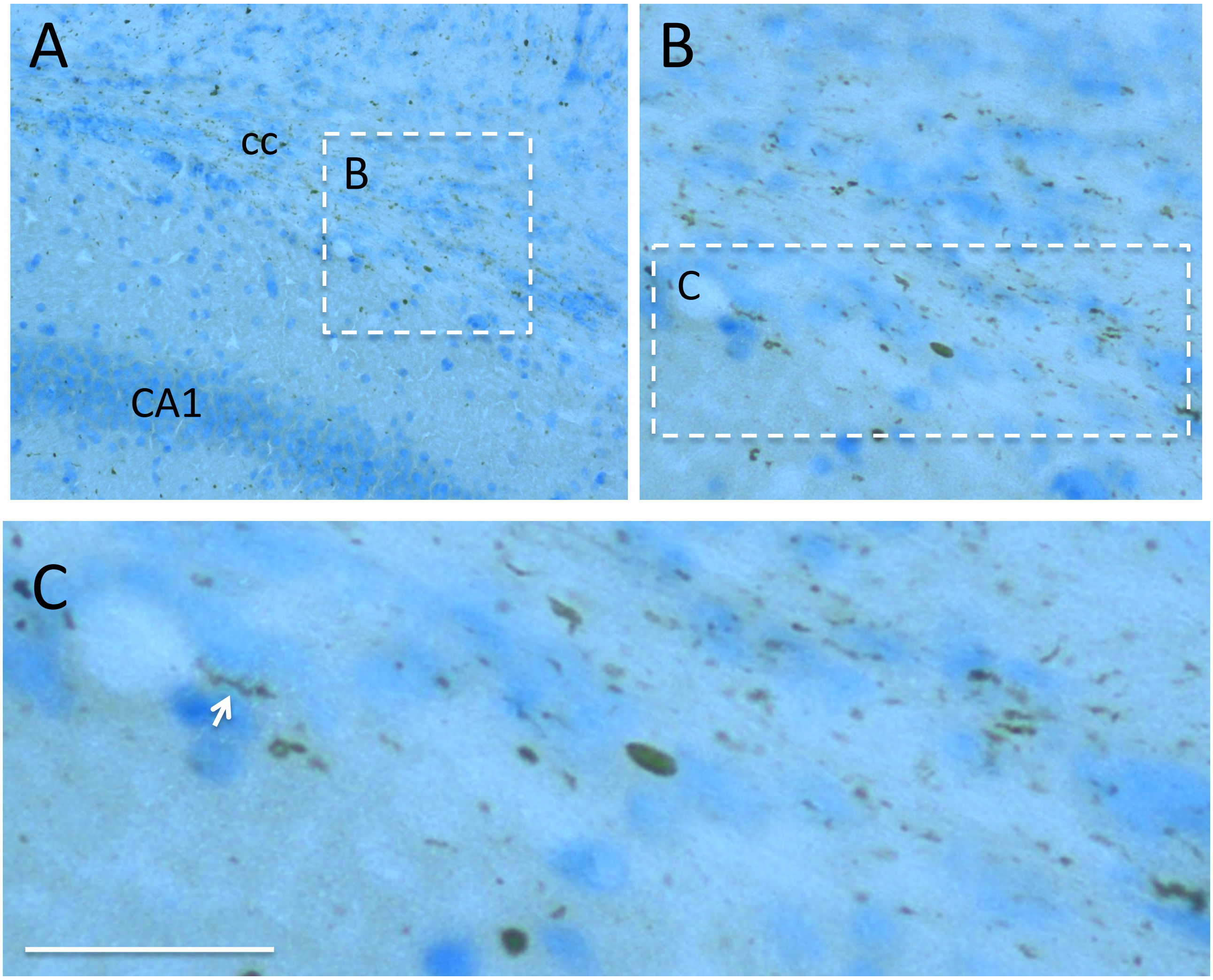

Supplementary Figure 9. White matter aggregates are present in corpus callosum of R6/2_50 mice at end stage. Photomicrographs show aggregates (brown staining) in corpus callosum but not CA1 of the hippocampus. Most aggregates are small and irregular, although occasional corkscrew aggregates similar to those seen in cerebellar white matter can be seen (arrowhead). The sections have been immunostained with MW8 and counterstained with methyl blue. Size bar in C=20µm. CA1= cornu Ammonis subfield 1; cc = corpus callosum
